# Tunable Chemical Grafting of Three-Dimensional Poly (3, 4-ethylenedioxythiophene)/Poly (4-styrenesulfonate)-Multiwalled Carbon Nanotubes Composite with Faster Charge-Carrier Transport for Enhanced Gas Sensing Performance

**DOI:** 10.3390/s20092470

**Published:** 2020-04-27

**Authors:** Hyojae Kim, Yeongseok Jang, Gyeong Won Lee, Seung Yun Yang, Jinmu Jung, Jonghyun Oh

**Affiliations:** 1 Department of Bio-Nano System Engineering, Jeonbuk National University, Jeonju 54896, Korea; hyojaekim@jbnu.ac.kr; 2 Department of Mechanical Design Engineering, Jeonbuk National University, Jeonju 54896, Korea; ysjang@jbnu.ac.kr; 3 Department of Biomaterials Science, Life and Industry Convergence Institute, Pusan National University, Miryang 50463, Korea; 22jungbi@gmail.com (G.W.L.); syang@pusan.ac.kr (S.Y.Y.); 4 Department of Nano-bio Mechanical System Engineering, Jeonbuk National University, Jeonju 54896, Korea; jmjung@jbnu.ac.kr

**Keywords:** carbon nanotubes, electrical properties, surface analysis, casting

## Abstract

The three-dimensional volumetric application of conductive poly (3,4-ethylenedioxythiophene)/poly (4-styrenesulfonate) (PEDOT:PSS) to multiwalled carbon nanotubes (MWCNTs) has not been widely reported. In this study, the applicability of the 3D PEDOT:PSS-MWCNT composite for a gas sensor was investigated with different PEDOT:PSS concentrations. The gas-sensing performance of the 3D PEDOT:PSS-MWCNT composites was investigated using ethanol and carbon monoxide (CO) gas. Overall, in comparison with the pristine MWCNTs, as the PEDOT:PSS concentration increased, the 3D PEDOT:PSS-MWCNT composites exhibited increased conductivity and enhanced gas sensing performances (fast response and recovery times) to both ethanol and CO gases. Importantly, although the PEDOT:PSS coating layer reduced the number of sites for the adsorption and desorption of gas molecules, the charge-carrier transport between the gas molecules and MWCNTs was significantly enhanced. Thus, PEDOT:PSS can be chemically grafted to MWCNTs to enhance the connectivity and conductivity of a 3D network, leading to possible applications in gas sensors.

## 1. Introduction

With the development of microfabrication technology and various materials, miniaturization studies on gas sensors have been proceeding rapidly. The miniaturization of the gas sensor created additional problems, including a reduced ability to adsorb and desorb gas molecules [1,2,3]. Therefore, to improve the performance of the miniaturized gas sensor, nanoscale materials such as carbon nanotubes with good mechanical and electrical properties were applied [4,5,6,7]. A variety of studies have been performed to facilitate the movement of electrons by forming functional groups on carbon nanotubes to adsorb specific gas molecules or bonding other substances to improve the electrical conductivity of carbon nanotubes [8,9].

Methods for forming various functional groups on carbon nanotubes were introduced to improve the binding affinity between carbon nanotubes and gas molecules, as summarized in Table 1.

Leghrib et al. reported on multiwalled carbon nanotubes (MWCNTs) decorated with tin oxide nanoclusters for the detection of nitrogen dioxide in a low-ppm range [10]. Sharma et al. employed a MWCNT/alumina (Al_2_O_3_) composite with a high response and reproducibility to NH_3_ [11]. Dhall et al. introduced acid-functionalized MWCNTs with fast response and recovery at room temperature for the detection of H_2_ gas [12]. Adjizian et al. presented boron- and nitrogen-doped MWCNT-based gas sensors for NO_2_, CO, C_2_H_4_, and H_2_O at ppm concentrations [13]. Abdulla et al. reported on the excellent gas-sensor properties of a polyaniline-functionalized MWCNT-based nanocomposite for the trace-level detection of ammonia (NH_3_) gas [14]. Cosio et al. used a glassy carbon electrode modified with MWCNTs functionalized with hydroxyl groups to investigate the electrochemical response of bisphenol A [15]. Zhang et al. developed a poly [3-(6-carboxyhexyl)thiophene-2,5-diyl] -functionalized single-walled carbon nanotube for the detection of methamphetamine vapor [16].

Additionally, studies have been performed to improve the electrical properties by increasing the conductivity of carbon nanotubes to improve the performance of gas sensors. Majumdar et al. introduced a nanosized CNT/SnO_2_ composite showing good performance at low resistance [17]. Ko et al. fabricated a WO_3_-coated MWCNT sensor with a high response to NO_2_ at room temperature [18]. Yaqoob et al. developed a MWCNT-WO_3_ nanoparticle hybrid with high NO_2_ sensing performance at room temperature [19]. Kwon et al. reported on the selective detection of a low concentration of toluene gas using platinum-decorated carbon nanotubes for increased sensing performance [20]. Eising et al. demonstrated composites based on carbon nanotubes and polyaniline films with a high response to ammonia [21]. Senocak et al. introduced a three-dimensional (3D) single-walled carbon nanotube material covalently functionalized with 1,6-diethynylpyrene that exhibited high response values to NH_3_ [22].

Despite considerable research, improvements in gas adsorption and electrical conductivity are challenging issues [23,24,25,26]. Water-soluble and conductive poly (3,4-ethylenedioxythiophene)/poly (4-styrenesulfonate) (PEDOT:PSS) is highly compatible with film-type gas sensing systems. In a two-dimensional PEDOT:PSS-based gas sensing system, gas molecules with very low density are adsorbed onto the surface of PEDOT:PSS film. Gas molecules adsorbed on film have little influence on the two-dimensional charge transport and conductivity, resulting in poor sensor response.

**Figure 1:**
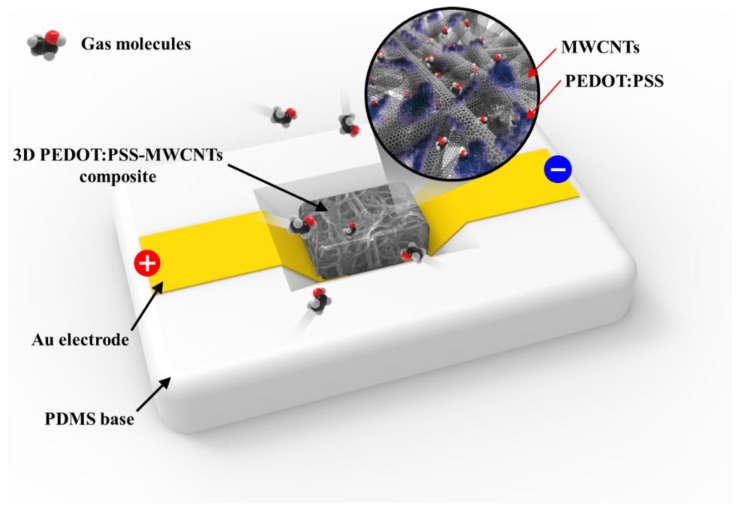
Schematic of gas-sensing module with 3D poly (3,4-ethylenedioxythiophene)/poly (4-styrenesulfonate) (PEDOT:PSS)- multiwalled carbon nanotubes (MWCNT) composite. PEDOT:PSS can be chemically grafted to MWCNTs to increase the strength and conductivity of the 3D network structure.

To overcome this problem, the carrier transport pathway can be improved using the three-dimensional structure of three kinds of conductive domains: PEDOT:PSS, MWCNT, and an interface between PEDOT:PSS and MWCNT [27]. Therefore, the conductivity of MWCNT composite conductors can be determined by the concentration of PEDOT:PSS. In this study, a 3D PEDOT:PSS-MWCNT composite was introduced for sensing gas molecules. The conductivity of the 3D PEDOT:PSS-MWCNT composite was investigated in terms of the gas response to different concentrations of PEDOT:PSS. PEDOT:PSS was chemically grafted onto MWCNTs, increasing the strength and conductivity of the 3D network structure, as shown in Figure 1.

Three types of 3D PEDOT: PSS-MWCNT composites were fabricated with different mixing ratios of MWCNTs and PEDOT:PSS. Structural analyses of the fabricated composites were performed using Raman spectroscopy and X-ray diffraction (XRD). The current distribution and topography over the composites were measured via conductive atomic force microscopy (cAFM). The morphology of the composites was investigated using scanning electron microscopy (SEM) and transmission electron microscopy (TEM). The electrical responses of the composites to ethanol and CO gases were demonstrated in a significantly low resistance range.

## 2. Materials and Methods

### 2.1. Fabrication of 3D PEDOT:PSS-MWCNT Composite

**Figure 2:**
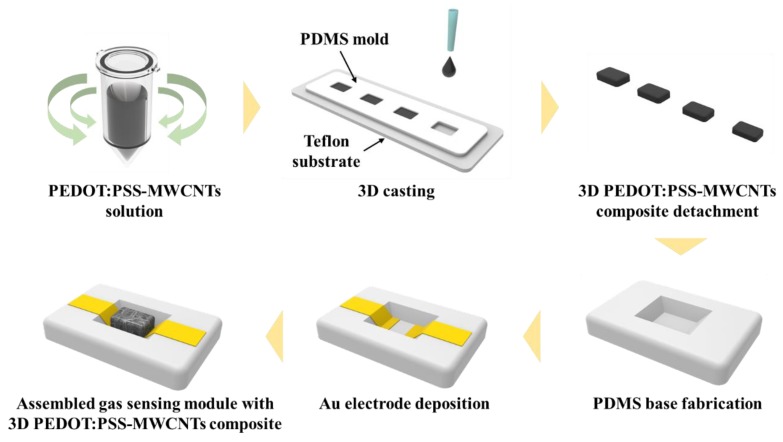
Fabrication process for the gas-sensing module using conductive 3D PEDOT:PSS-MWCNT composite.

Figure 2 schematically depicts the fabrication process for the 3D PEDOT:PSS-MWCNT composites. A PEDOT:PSS (Clevios PH 1000, Heraeus Holding GmbH, Hanau, Germany) aqueous solution and pristine MWCNTs (CNT MR99, Carbon Nano-material Technology Co., Ltd., Pohang, Korea) were purchased. With 0.01 g of CNTs, 0.5, 1, and 5 wt% of PEDOT:PSS was added to deionized water. The mixed solution was stirred using a vortex mixer (Vortex-Genie 2, Scientific Industries, Inc, Bohemia, NY, USA) under 2800 rpm for 30 min. A polydimethylsiloxane (PDMS) kit (Sylgard 184, Dow Corning, Midland, MI, USA) was used to prepare a mold for casting 3D PEDOT:PSS-MWCNT composites. Prepolymer resin and a curing agent were mixed and stirred at a ratio of 10:1. After a degassing process, the mixture was cured at 85 °C for 2 h. The size of the PDMS mold was 12 (width) × 8 (depth) × 1 (height) mm^3^. The prepared PDMS mold was attached to the surface of a polytetrafluoroethylene (PTFE) substrate. Then, 300 µL of the PEDOT:PSS-MWCNT solution was poured into the PDMS mold and dried in an oven at 40 °C for 18 h. The dried 3D PEDOT:PSS-MWCNT composite was then detached from the PTFE substrate without any deformation. 

The mold for the sensor base (50 × 30 × 3 mm) was printed using a 3D stereolithography printer (Form2, Formlabs Inc., Somerville, MA, USA). The PDMS prepolymer solution was poured into the 3D-printed mold and cured at 85 ℃ for 2 h in an oven. The fabricated PDMS mold for the sensor base was then patterned using a thermal tape for gold electrode deposition. Using an electron-beam evaporation process, a 20-nm-thick titanium layer and 200-nm-thick gold layer were deposited on the patterned PDMS base. To fabricate the sensor module for the gas experiment, the 3D PEDOT:PSS-MWCNT composite was permanently bonded to the gold electrode using silver paste [27,28].

### 2.2. Characterization of 3D PEDOT:PSS-MWCNT Composites

The effect on the chemical grafting of the PEDOT:PSS to the MWCNTs was investigated via Raman spectroscopy and XRD. Raman spectroscopy (LabRam GR UV/Vis/NIR, Horiba Ltd., Kyoto, Japan) was performed using 514-nm laser excitation. XRD (X’pert Pro Power, PANalytical, Almelo, Netherlands) was performed at an angle of 2θ. The morphology of the PEDOT:PSS-coated MWCNTs was analyzed using ultrahigh-resolution field-emission SEM (UHR-FE-SEM, S-5500, Hitachi Ltd., Chiyoda, Japan) and field-emission energy-filtered TEM (FE-EF-TEM, JEM-2200FS, JEOL Ltd., Akishima, Japan). The samples for UHR-FE-SEM did not require any coating for image acquisition. 

To obtain the FE-EF-TEM images, samples were prepared by dropping the composite on a Lacey carbon grid at a voltage of 200 kV. The coated condition of PEDOT:PSS was also investigated using energy-dispersive X-ray spectroscopy (EDS). The electrical conductivity was measured by obtaining the current-voltage (I-V) curves using cAFM. The cAFM analysis was performed in the contact mode. The scan sizes were 500 nm × 500 nm, and the scan frequency was 0.5 Hz. A platinum-coated tip was used to probe the samples in the bias range of −3 to +3 V.

### 2.3. Testing of Gas-Sensing Performance

The gas sensing properties of 3D PEDOT:PSS-MWCNT composites were investigated using a two-probe connection with a source meter (Keithley 2400, Tektronix, Inc., Beaverton, OR, USA). The gas flow and chamber pressure were controlled by a mass flow controller (MFC), and the concentrations of ethanol and CO were generated by using nitrogen (N_2_) gas as a diluting gas. The relative humidity was maintained below 8% during the experiment. All experiments were initiated at a chamber pressure of 10 mTorr, constant bias voltage of 5 V, and room temperature (25 °C). Ethanol and CO gas were injected into the sensing chamber at a pressure of 250 mTorr (see Figure S1). The response (S) is defined as the ratio *S* = ∆*R*/*R* × 100%, where *R* represents the initial resistance and ∆*R* represents the resistance variation after gas injection. Each pristine, 0.5, 1, and 5 wt% PEDOT:PSS-coated MWCNT sample was prepared in three replicates using the same fabrication process. The test for each sample was repeated three times. The data are presented as mean ± standard deviation (SD) for each sample.

## 3. Results and Discussion

**Figure 3:**
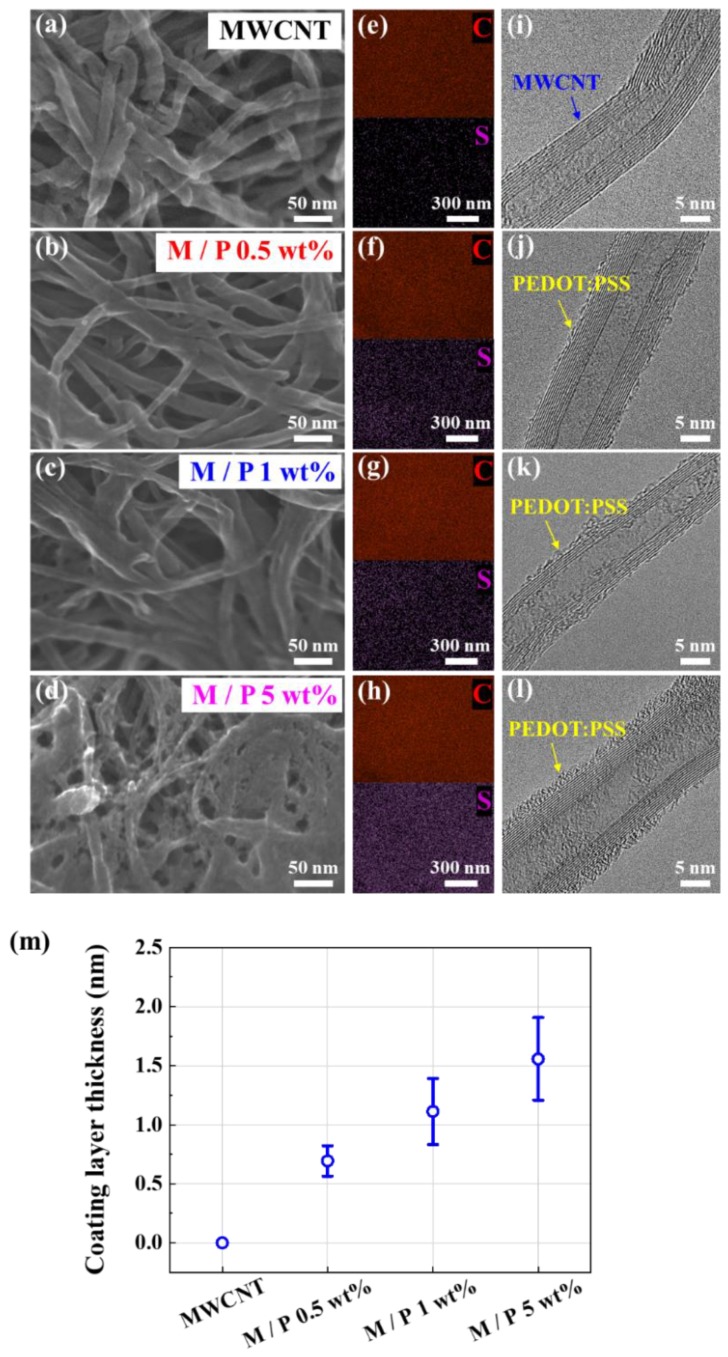
SEM images of (**a**) pristine MWCNTs and MWCNTs coated with (**b**) 0.5 wt% PEDOT-PSS, (**c**) 1 wt% PEDOT-PSS, and (**d**) 5 wt% PEDOT-PSS. EDS mapping for (**e**) pristine MWCNTs and MWCNTs coated with (**f**) 0.5 wt% PEDOT-PSS, (**g**) 1 wt% PEDOT-PSS, and (**h**) 5 wt% PEDOT-PSS. TEM images of (**i**) pristine MWCNTs and MWCNTs coated with (**j**) 0.5 wt% PEDOT-PSS, (**k**) 1 wt% PEDOT-PSS, and (**l**) 5 wt% PEDOT-PSS. (**m**) a coating layer thickness of PEDOT:PSS on the outer walls of MWCNTs.

Figure 3 shows the UHR-FE-SEM, EDS, and FE-EF-TEM images for both pristine MWCNTs and the 3D PEDOT:PSS-MWCNT conductive composite. Figure 3a shows the pristine MWCNTs without the PEDOT:PSS coating. Compared with Figure 3a, Figure 3b–d clearly shows that the PEDOT:PSS covered the outer wall of the MWCNTs. With an increasing concentration of the PEDOT:PSS binder, a larger amount of PEDOT:PSS was involved in the structural networks between MWCNTs. EDS mapping was performed to investigate the distribution of PEDOT:PSS on the outer wall of the MWCNTs. As shown in Figure 3e–h, the element sulfur, representing PEDOT:PSS, was homogeneously distributed on the MWCNTs. 

With an increased coating concentration of PEDOT:PSS, a higher intensity of sulfur was observed, thus helping to confirm the coated amount of PEDOT:PSS in Figure 3b–d. Figure 3i shows a TEM image of an individual pristine MWCNT that exhibited a regular morphology. In Figure 3j–l, the attached PEDOT:PSS is observed on the outer walls of MWCNTs with different thicknesses depending on the coating level. The average thickness of the PEDOT:PSS attached to the outer walls of the MWCNTs was measured, as shown in Figure 3m. When the PEDOT:PSS concentration was changed to 0.5, 1, and 5 wt%, the average thickness increased linearly to 0.69 ± 0.13, 1.11 ± 0.28, and 1.56 ± 0.35 nm, respectively. These results confirm the important role of PEDOT:PSS in connecting MWCNTs to generate 3D conductive structural networks.

**Figure 4:**
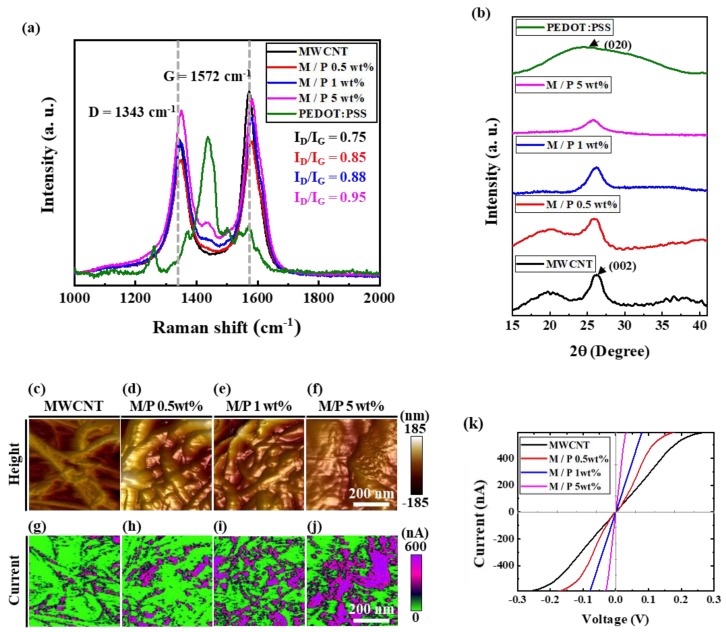
(**a**) Raman and (**b**) XRD spectra of pristine MWCNTs and MWCNTs coated with 0.5, 1, and 5 wt% PEDOT:PSS. Topographic images of (**c**) pristine MWCNTs and MWCNTs coated with (**d**) 0.5 wt%, (**e**) 1 wt%, and (**f**) 5 wt% PEDOT:PSS. Current mapping for (**g**) pristine MWCNTs and MWCNTs coated with (**h**) 0.5 wt%, (**i**) 1 wt%, and (**j**) 5 wt% PEDOT:PSS. (**k**) I-V curves for pristine and 0.5 wt%, 1 wt%, and 5 wt% PEDOT:PSS-coated MWCNTs.

Figure 4a shows Raman spectroscopic data for 3D composites according to PEDOT:PSS concentration. The Raman spectra indicate the typical D band around 1343 cm^−1^ and a G band around 1572 cm^−1^. As the PEDOT:PSS concentration in the 3D composite increased, the I_D_/I_G_ ratios approached 1, indicating the existence of many defects in the pristine MWCNTs. These phenomena show that the PEDOT:PSS was chemically grafted onto the surface of the MWCNTs. Compared with the peak for the pristine MWCNTs, the peaks for the 3D composites were blue-shifted from 1343 to 1350 cm^−1^ owing to the increase in the PEDOT:PSS concentration.

Figure 4b shows the XRD patterns for samples with different mixing ratios of MWCNTs and PEDOT:PSS. PEDOT:PSS at 24.8° shows a broad and slight peak corresponding to the (020) plane of the backbone of the PEDOT:PSS. As the PEDOT:PSS concentration increased, the typical sharp peak of the MWCNTs became flat because of their reduced crystallinity.

Figure 4c–k depict the electrical properties of the samples, which were highly influenced by the concentration of PEDOT:PSS. The electrical properties contribute to the charge-carrier transport through the 3D network structure in the gas-sensing mechanism. Figure 4c–f illustrate topographic images obtained via cAFM for PEDOT:PSS concentrations of 0, 0.5, 1, and 5 wt%, respectively. With an increase in the PEDOT:PSS concentration, the PEDOT:PSS layer covered a wider area on the surface of the MWCNTs, and the topographic peaks were reduced. As shown in Figure 4g–j, the current maps obtained via cAFM exhibited a different pattern from that of the topographic images. As the PEDOT:PSS concentration increased, the peaks of the purple area (indicating a high current) were distributed evenly. This phenomenon can be attributed to the decreased space between the nanotubes, resulting in increased network conductivity.

Figure 4k presents the I-V curves of samples with different PEDOT:PSS concentrations when measured at room temperature. The MWCNTs coated with PEDOT:PSS showed ohmic behavior within the voltage range of −0.3 to 0.3 V. The linear ohmic behavior indicates the low-resistance flow of charge carriers through the MWCNTs coated with PEDOT:PSS. However, the slopes differ among the samples according to the PEDOT:PSS concentration. The slope of the I-V curve increased with the PEDOT:PSS concentration, indicating a higher conductivity than the pristine MWCNTs.

**Figure 5:**
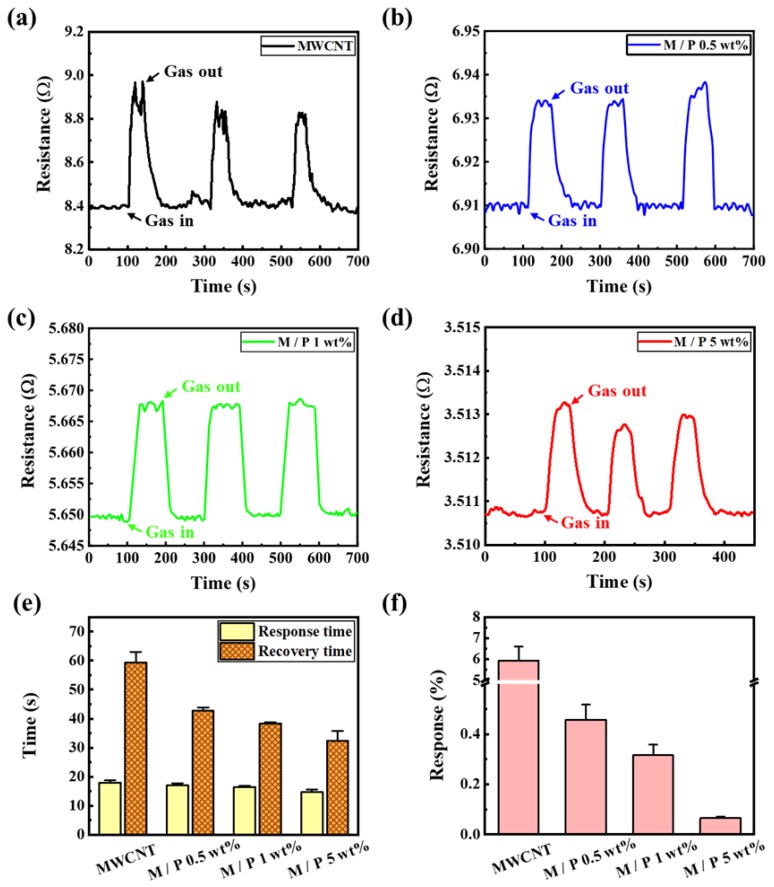
Response curves to 400 ppm of ethanol gas for (**a**) pristine MWCNTs and (**b**) 0.5 wt% PEDOT:PSS-MWCNTs. (**c**) 1 wt% PEDOT:PSS-MWCNTs. (**d**) 5 wt% PEDOT:PSS-MWCNTs. (**e**) Response and recovery times of samples to 400 ppm ethanol gas at room temperature (**f**) Response differences among pristine and 0.5 wt%, 1 wt%, and 5 wt% PEDOT:PSS-coated MWCNTs.

Figure 5a–d show the response curves of the pristine MWCNTs, 0.5, 1, and 5 wt%-PEDOT:PSS-coated MWCNTs to 400 ppm of ethanol gas at room temperature, respectively. Although both curves indicate repeated stable adsorption and desorption, the resistance range was reduced as the PEDOT:PSS concentration increased. For example, the resistance range of 8.395–8.965 Ω for the pristine MWCNTs was reduced to 3.510–3.514 Ω for the 5 wt% PEDOT:PSS-coated MWCNTs. This reduction in the base resistance could be caused by the enhanced conductivity of the sample due to filling and then connecting the space between the MWCNTs with the PEDOT:PSS polymer. In addition, we measured the signal-to-noise ratio (SNR) of each sample for ethanol. The SNR values of the pristine MWCNTs, 0.5, 1, and 5 wt% PEDOT:PSS-coated MWCNTs were 7.1, 40.1, 43.2, and 46.4, respectively. The SNR value tended to increase with an increased concentration of PEDOT: PSS.

Moreover, in Figure 5e, compared with the pristine MWCNTs, the response time was reduced from 18.0 ± 0.8 to 14.6 ± 0.9 s, and the recovery time significantly improved from 59.3 ± 8.7 to 32.3 ± 4.5 s, owing to the effect of the PEDOT:PSS coating. Figure 5f shows the response to ethanol gas with different levels of PEDOT:PSS coating on the MWCNTs. The response was calculated (*S* = Δ*R*/*R* × 100%) as 5.93 ± 0.65, 0.45 ± 0.06, 0.31 ± 0.041, and 0.065 ± 0.005 for the pristine MWCNTs and 0.5 wt%, 1 wt%, and 5 wt% PEDOT:PSS-coated MWCNTs, respectively. Interestingly, the concentration of 5 wt% PEDOT:PSS exhibited the best conductivity and the fastest response to gas sensing.

The response of the 5 wt% PEDOT:PSS-coated MWCNTs under various concentrations of ethanol was proportionally reduced as the concentration of ethanol was decreased (see Figure S2a). This phenomenon is explained as follows: as the outer walls of the MWCNTs were covered by the PEDOT:PSS coating, the conductivity could be enhanced. Although the number of sites for gas-molecule adsorption was reduced by the PEDOT:PSS coating, the charge transfer of the gas molecules absorbed in the reduced sites of MWCNTs could be enhanced through a highly conductive PEDOT:PSS surface, resulting in an improved response to the gas molecules of the 3D conductive PEDOT:PSS-MWCNT composite in comparison with the pristine MWCNTs.

**Figure 6:**
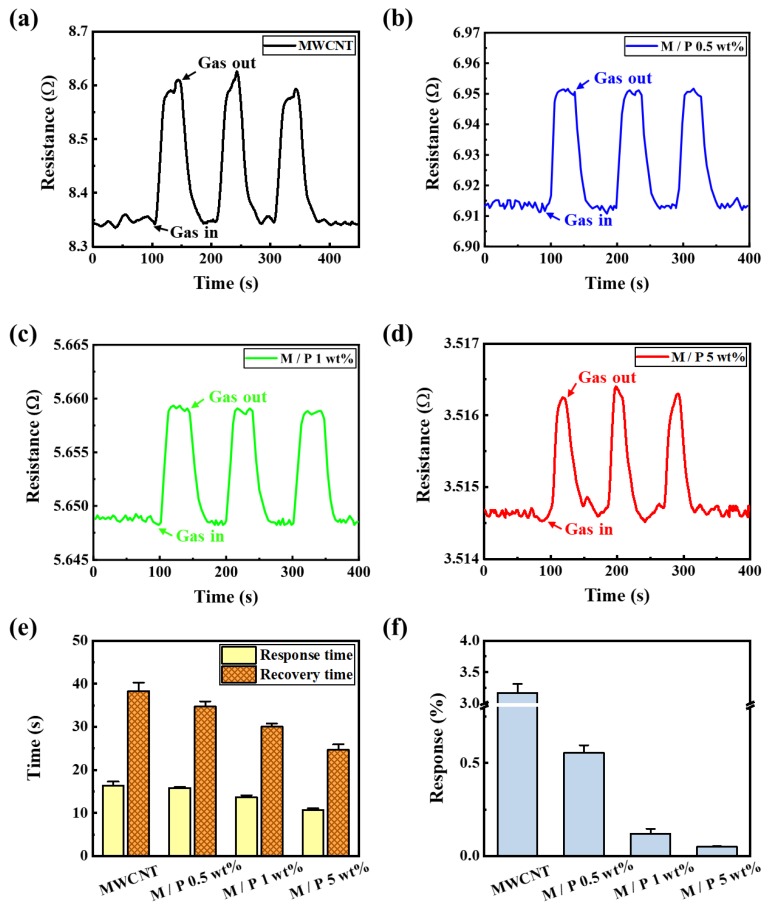
Response curves to 1000 ppm of CO gas for (**a**) pristine MWCNTs and (**b**) 0.5 wt% PEDOT:PSS-MWCNTs. (**c**) 1 wt% PEDOT:PSS-MWCNTs. (**d**) 5 wt% PEDOT:PSS-MWCNTs. (**e**) Response and recovery times of samples to 1000 ppm CO gas at room temperature (**f**) Response characterization for pristine and 0.5 wt%, 1 wt%, and 5 wt% PEDOT:PSS-coated MWCNTs.

Figure 6 shows the reproducibility and response of the pristine MWCNTs, 0.5, 1, and 5 wt% PEDOT:PSS-coated MWCNTs to 1000 ppm of CO gas at room temperature. As shown in Figure 6a, the pristine MWCNTs exhibited stable repeatability and response with a resistance range of 8.33–8.62 Ω. As shown in Figure 6b–d, the resistance of the PEDOT:PSS-coated MWCNTs decreased to 3.5165 Ω. Additionally, the SNR values of the pristine MWCNTs, 0.5, 1, and 5 wt% PEDOT:PSS-coated MWCNTs were 12.2, 36.4, 36.5, and 54.5, respectively.

Similar to the results of Figure 5e, in Figure 6e, the response time for CO gas improved from 16.3 ± 0.9 to 10.6 ± 0.4 s, and the recovery time for CO gas was reduced from 38.3 ± 1.9 to 24.6 ± 1.2 s, owing to the PEDOT:PSS coating. For PEDOT:PSS concentrations of 0, 0.5, 1, and 5 wt%, the response of the PEDOT:PSS-coated MWCNTs was 3.17 ± 0.146, 0.55 ± 0.042, 0.12 ± 0.026, and 0.05 ± 0.004, respectively (Figure 6f). The 5 wt% PEDOT:PSS-coated MWCNTs also showed a proportional increase in response with an increasing concentration of CO gases from 250 to 1000 ppm (see Figure S2b). 

The sensing mechanism of PEDOT:PSS-MWCNT is based on changes in electrical conductivity caused by the adsorption of gas molecules (ethanol and CO). The holes in the conductive PEDOT:PSS-MWCNT can be depleted by electrons donated from the gas molecules [29]. Since PEDOT:PSS-MWCNT behaves as a p-type semiconductor, the depleted holes can decrease the sensitivity upon gas exposure, leading to a reduction in the sensor response. The electrical conductivity can be improved by forming more conductive channels in the 3D PEDOT:PSS-MWCNT matrix by increasing the concentration of conductive PEDOT:PSS polymer, resulting in enhanced gas sensing performance (fast response time and recovery time) [30,31,32]. These beneficial electrical properties indicate that the 3D conductive PEDOT:PSS-MWCNT composite can be useful for fast gas sensing at room temperature in various concentration environments.

In this study, we tried to enhance the adsorption ability of gas molecules through the increased surface area by creating a three-dimensional conductive structure of the sensor. Fast response and recovery time could be achieved through a three-dimensional conductive network by using the fabricated three-dimensional structure of the PEDOT:PSS-coated MWCNT composite. However, despite the more increased surface area than the previously reported two-dimensional PEDOT:PSS-based gas sensor, the response of the 3D PEDOT:PSS-MWCNT composite was not sufficiently enhanced. This might be caused by the reduced number of adsorption sites of MWCNTs by PEDOT:PSS coating layer, which remains as a challenging work. 

## 4. Conclusions

A 3D PEDOT:PSS-MWCNT composite was introduced for sensing gas molecules. Three types of 3D PEDOT:PSS-MWCNT composites were fabricated with different concentrations of PEDOT:PSS. The material and electrical properties were analyzed using Raman spectroscopy, XRD, cAFM, SEM, and TEM. The performance of the fabricated composites was investigated using ethanol and CO gas at room temperature. The chemical grafting of PEDOT:PSS to the MWCNTs was confirmed by Raman and XRD spectra. In the SEM and TEM images, the PEDOT:PSS layer coated on the outer walls of the MWCNTs was clearly observed, revealing the structural networks between MWCNTs. The cAFM results revealed that the PEDOT:PSS-coated MWCNTs exhibited higher conductivity than the pristine MWCNTs. 

The linear ohmic behavior indicated the low-resistance flow of charge carriers through the MWCNTs coated with PEDOT:PSS. The reproducibility and response of the 3D PEDOT:PSS-MWCNT composites were characterized for both ethanol and CO gases. Overall, the 3D PEDOT:PSS-MWCNT composites exhibited increased conductivity and enhanced gas sensing performances to both ethanol and CO gases as the PEDOT:PSS concentration increased. Importantly, although the PEDOT:PSS coating layer reduced the number of sites for the adsorption and desorption of gas molecules, the charge-carrier transport between the gas molecules and MWCNTs was significantly enhanced owing to the use of PEDOT:PSS. Thus, the application of PEDOT:PSS to MWCNT-based gas sensors can be invaluable for fast sensing performance in various concentration environments.

## Figures and Tables

**Table 1:** Analytical performance comparison of previously reported works with carbon nanotubes (CNT) gas sensor.

Materials	Fabrication Method	Targeted Analyte	Working Temperature (°C)	Recovery Time (s)	Ref.
CNT/SnO_2_	Oxidative functionalization	CO	RT	-	[10]
MWCNT/Al_2_O_3_	Acid treatment	NH_3_	RT	-	[11]
F-MWCNTs	Acid treatment	H_2_	RT	100	[12]
N-MWCNTs	Film deposition	NO_2_	150	2400	[13]
PANI/MWCNTs	Oxidative polymerization	NH_3_	RT	35–62	[14]
MWCNTs-COOH	Chemical modification	Bisphenol A	RT	10	[15]
P3CT/CNTs	Drop-casting	NMPEA	RT	40	[16]
CNT/SnO_2_	Wet chemical method	H_2_	200	>120	[17]
CNT/WO_3_	Sputter deposition	NO_2_	RT	>300	[18]
MWCNTs-WO_3_NPs	Hydrothermal synthetization	NO_2_	RT	1620	[19]
Pt-MWCNTs	Sputter deposition	C_7_H_8_	150	>70	[20]
PANI:CNTs	Interfacial polymerization	NH_3_	RT	46	[21]
SWCNTs-Pyrene 3D Hybrid	Drop-casting	NH_3_	22	20	[22]

* RT = Room temperature; F-MWCNTs = acid-functionalized MWCNTs; N-CNTs = nitrogen-doped MWCNT; PANI = Polyaniline; P3CT = poly [3-(6-carboxyhexyl)thiophene-2,5-dity]; NMPEA = N-Methylphenethylamine.

## Supplementary Materials

The following are available online at https://www.mdpi.com/1424-8220/20/9/2470/s1, Figure S1: Schematic of ethanol and CO gas testing setup to characterize 3D PEDOT:PSS-coated MWCNTs gas sensing system. The specific concentration of ethanol and CO gases was precisely controlled by mixing dry N_2_ at different ratios, and mixed gas was then injected into the sensing chamber. Resistance from the gas sensor was measured with a two-probe connection using a sourcemeter. Figure S2: Response curves of ethanol and CO gas for 5 wt% PEDOT:PSS-MWCNTs depending on concentration.
