# Supplementary material for: Tunable Chemical Grafting of Three-Dimensional Poly (3, 4-ethylenedioxythiophene)/Poly (4-styrenesulfonate)-Multiwalled Carbon Nanotubes Composite with Faster Charge-Carrier Transport for Enhanced Gas Sensing Performance
Source: Sensors (Basel). 2020 Apr 27;20(9):2470. doi: 10.3390/s20092470 (PMC7250038; doi:10.3390/s20092470)
Supplement: Supplementary file 1 [file sensors-20-02470-s001.pdf]

sensors

Article

Tunable Chemical Grafting of Three-Dimensional Poly (3, 4-ethylenedioxythiophene)/Poly (4-styrenesulfonate)-Multiwalled Carbon Nanotubes Composite with Faster Charge-Carrier Transport for Enhanced Gas Sensing Performance

Hyojae Kim [1,†], Yeongseok Jang [2,†], Gyeong Won Lee [3,†], Seung Yun Yang [3,†], Jinmu Jung [4,†] and Jonghyun Oh [4,*]

1 Department of Bio-Nano System Engineering, Jeonbuk National University, Jeonju 54896, Korea; hyojaekim@jbnu.ac.kr
2 Department of Mechanical Design Engineering, Jeonbuk National University, Jeonju 54896, Korea; ysjang@jbnu.ac.kr
3 Department of Biomaterials Science, Life and Industry Convergence Institute, Pusan National University, Miryang 50463, Korea; 22jungbi@gmail.com (G.W.L), syang@pusan.ac.kr (S.Y.Y)
4 Department of Nano-bio Mechanical System Engineering, Jeonbuk National University, Jeonju 54896, Korea; jmjung@jbnu.ac.kr (J.J), jonghyuno@jbnu.ac.kr (J.O)
* Correspondence: jonghyuno@jbnu.ac.kr
† These authors contributed equally to this work

Received: 05 April 2020; Accepted: 24 April 2020; Published: date

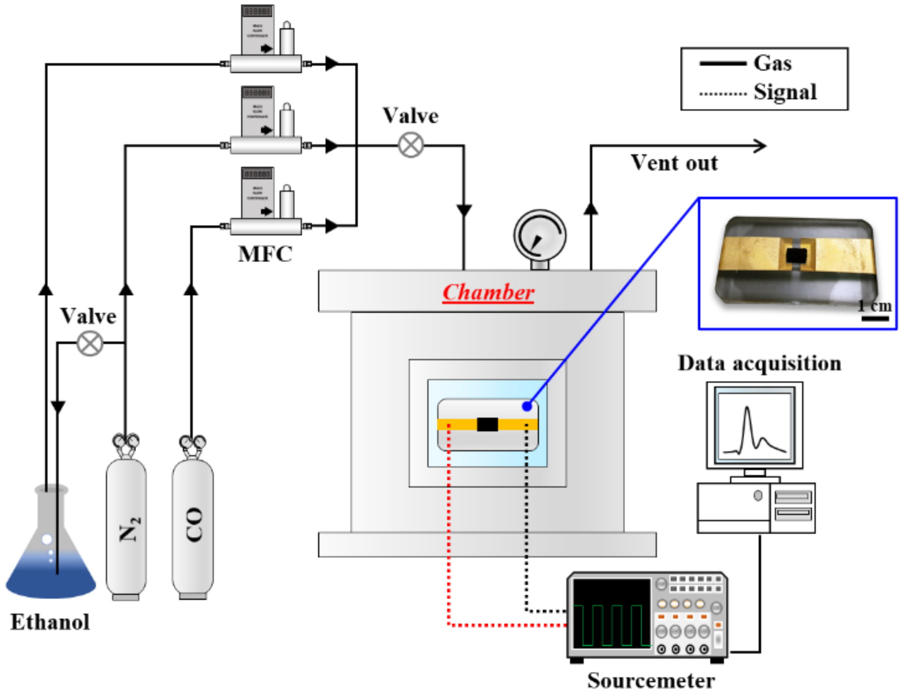

Figure S1. Schematic of ethanol and CO gas testing setup to characterize 3D PEDOT:PSS-coated MWCNTs gas sensing system. The specific concentration of ethanol and CO gases was precisely controlled by mixing dry N_2 at different ratios, and mixed gas

was then injected into the sensing chamber. Resistance from the gas sensor was measured with a two-probe connection using a sourcemeter.

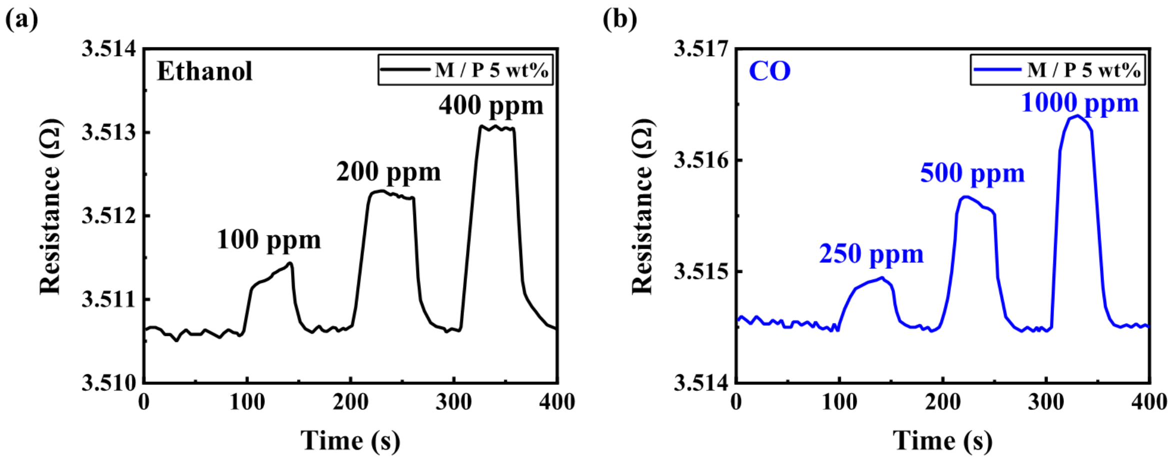

Figure S2. Response curves of ethanol and CO gas for 5 wt% PEDOT:PSS-MWCNTs depending on concentration.

© 2020 by the authors. Submitted for possible open access publication under the terms and conditions of the Creative Commons Attribution (CC BY) license (http://creativecommons.org/licenses/by/4.0/).
